# Search for New Compounds with Anti-Inflammatory Activity Among 1,2,4-Triazole Derivatives

**DOI:** 10.3390/molecules29246036

**Published:** 2024-12-21

**Authors:** Teresa Glomb, Julia Minta, Michalina Nowosadko, Julia Radzikowska, Piotr Świątek

**Affiliations:** 1 Department of Medicinal Chemistry, Faculty of Pharmacy, Wroclaw Medical University, Borowska 211, 50-556 Wrocław, Poland; teresa.glomb@umw.edu.pl; 2 Student Science Club of Medicinal Chemistry, Department of Medicinal Chemistry, Faculty of Pharmacy, Wroclaw Medical University, Borowska 211, 50-556 Wrocław, Poland; julia.minta@student.umw.edu.pl (J.M.); michalina.nowosadko@student.umw.edu.pl (M.N.); julia.radzikowska@student.umw.edu.pl (J.R.)

**Keywords:** 1,2,4-triazole, anti-inflammatory activity, cyclooxygenase inhibitors, lipoxygenase inhibitors, activity in vitro, activity in vivo

## Abstract

Compounds containing the 1,2,4-triazole moiety in their structure exhibit broad biological activities. Many of these compounds demonstrate anti-inflammatory activity in vitro through various mechanisms, such as inhibiting COX-1/COX-2 and LOX, modulating pro-inflammatory cytokine levels, or having effects on other specific enzymes. Some also display activities in vivo. In many publications, the activities of new 1,2,4-triazole-based compounds exceed those of the reference drugs, suggesting their promising potential as new therapeutic agents. This review of active 1,2,4-triazole derivatives with anti-inflammatory activity is based on literature published from 2015–2024.

## 1. Introduction

Triazoles are five-membered heterocyclic scaffolds containing in their structure three nitrogen atoms and two carbon atoms. In the case of the position of the nitrogen atoms, we can differentiate two isomeric forms of triazole, 1,2,4-triazole and 1,2,3-triazole. Each displays tautomers determined by the hydrogen bonded to the nitrogen in the ring. Considering the 1,2,4-triazole, the 1*H* tautomer is more stable than the 4*H* form [1].

**Figure 1:**
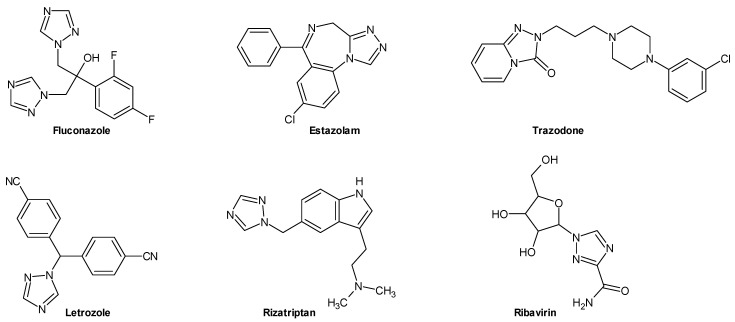
Examples of known drugs containing a 1,2,4-triazole ring.

The 1,2,4-triazole ring occurs in the structure of many drugs, e.g., antifungal fluconazole, anxiolytic estazolam, antidepressant trazodone, aromatase inhibitor letrozole, antimigraine rizatriptan, and antiviral ribavirin (Figure 1).

A literature review showed that there are many methods for synthesizing triazole derivatives [1,2,3]. The existence of the 1,2,4-triazole ring influences the pharmacokinetic and physicochemical abilities of the full structure. The 1,2,4-triazole ring as a significant pharmacophore behaves as an isostere of carboxylic acid, amide, ester, and alternative heterocycles such as pyrazole. The valuable attributions of the increased biological activities of the 1,2,4-triazole ring contain the ability to create hydrogen bonds and interactions with the receptors with high affinity as a result of their dipole character, and they have high chemical stability, rigidity, and solubility [4,5].

The above details of the 1,2,4-triazole ring have resulted in a large diversity of pharmaceutical applications for this pharmacophore. Based on current literature, scientists all over the world have been synthesizing new molecules bearing the 1,2,4-triazole core, which has exhibited a broad range of biological effects, including cytotoxic [6,7], antioxidant [8], or antimicrobial activities on bacteria [9,10], fungi [11,12], and viruses [13].

Moreover, the literature confirms the wide anti-inflammatory activity of compounds which include the 1,2,4-triazole ring in their structure.

Considering the comprehensive anti-inflammatory activity of 1,2,4-triazole derivatives, in this review, we aimed to compile the accomplishments of experts in medicinal chemistry and other scientists in recent years. In generally analyzing the structure of the derivatives included in this literature review, it could be seen that they are dominated by derivatives containing a 1,2,4-triazole ring with a free thiol or thione group or their *S*-substituted structures. This may be due to the ease of obtaining such scaffolds in the cyclization process from thiosemicarbazide and substrate availability [1]. Of course, many derivatives without a sulfur atom are also included in this review. The properties of derivatives in which the 1,2,4-triazole ring is fused with other heterocyclic rings or connected to a steroid residue are also discussed.

This review refers to publications from 2015 to 2024, and in our literature search, the PubMed database was used by entering the keywords “anti-inflammatory”, “activity”, and “1,2,4-triazole”. In the selected works, the most active compounds were divided due to their mechanism of action.

## 2. Anti-Inflammatory Activity of 1,2,4-Triazole Derivatives

### 2.1. 1,2,4-Triazole Derivatives as COX-1/COX-2 Inhibitors

Inflammation is the body’s defense response, but chronic inflammation can lead to cardiovascular diseases and cancer. Cyclooxygenase (COX) enzymes catalyze the biosynthesis of active pro-inflammatory mediators like prostaglandins from arachidonic acid (AA). There are at least two isoforms of COX—the constitutive COX-1 and the inflammatory-activated COX-2. Researchers have been searching for substances that can block, in particular, the COX-2 enzyme which inhibits the formation of pro-inflammatory prostaglandins and reduces the development of inflammation [14]. Examples of COX inhibitors, including 1,2,4-triazole derivatives, are described below.

**Figure 2:**
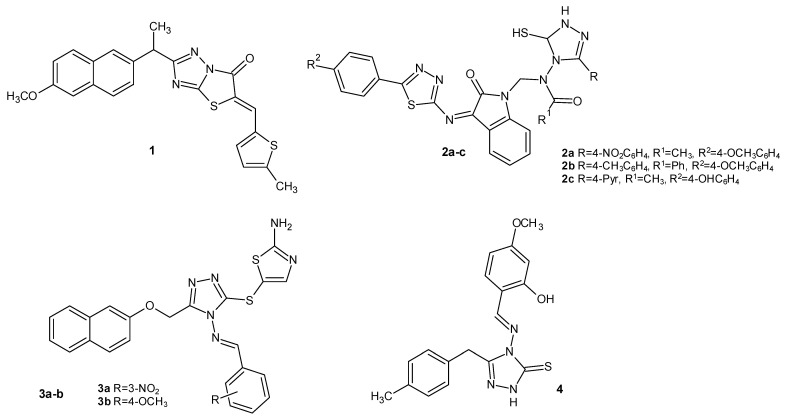
1,2,4-triazole derivatives as COX inhibitors (part 1).

Sarigol’s team (2015) modified the naproxen molecule, developed a new series of thiazole [3,2-*b*]-1,2,4-triazoles, and assessed their anti-inflammatory effects. Compound **1** (Figure 2) was the most selective COX-2 inhibitor (COX-1 IC_50_ > 100 µM and COX-2 IC_50_ = 20.5 µM) of all the synthesized derivatives, and it turned out to be more potent than the reference drug indomethacin (COX-1 IC_50_ = 0.65 µM and COX-2 IC_50_ = 29.6 µM). Its anti-inflammatory effect was also confirmed in vivo in the carrageenan-induced paw edema test. The most effective compound **1** displayed a significantly lower risk of ulcers than the standard drugs indomethacin and naproxen, which may have been related to the substitution of the free carboxylic acid group in the naproxen with a thiazole [3,2-*b*]-1,2,4-triazole ring [15].

In vitro anti-inflammatory studies on 1,2,4-triazole-conjugated 1,3,4-thiadiazole hybrid synthesized by Pathak et al. in 2020 showed that compounds **2a**, **2b**, and **2c** (Figure 2) were the strongest cyclooxygenase inhibitors in the series of structures tested compared to the standard drug, celecoxib (COX-1 inhibition of approximately 50%, COX-2 inhibition of approximately 70%). The results were also confirmed in vivo, and the compounds showed similar activity for diclofenac in the carrageenan-induced paw edema model. Additionally, the **2b** structure reduced the levels of pro-inflammatory mediators such as tumor necrosis factor (TNF-α) and various interleukins (IL-6 and IL-1) [16].

In 2020, researchers under the direction of Elrayess designed and synthesized a series of 1,2,4-triazole Schiff bases and evaluated them as potent and selective COX-2 inhibitors. Compounds **3a** and **3b**, presented in Figure 2, were the strongest of the designed compounds in vitro (COX-1 IC_50_ = 12.47–13.0 µM and COX-2 IC_50_ = 0.04 µM), and they were more potent than the reference drug, celecoxib (COX-1 IC_50_ = 14.7 µM and COX-2 IC_50_ = 0.05 µM) [14].

In a 2020 study, Munir et al. developed a series of Schiff bases bearing a 1,2,4-triazole ring and examined their anti-inflammatory activity. Among them, derivative 4 (Figure 2), with IC_50_ values of 117.8 µM (COX-1) and 1.76 µM (COX-2), emerged as a potent and selective COX-2 inhibitor in vitro in comparison to indomethacin (COX-1 IC_50_ = 0.25 µM and COX-2 IC_50_ = 0.07 µM) and diclofenac (COX-1 IC_50_ = 0.48 µM and COX-2 IC_50_ = 10.05 µM). However, in vivo, it showed weaker effects on carrageenan-induced paw edema in mice than the reference drug, indomethacin [17].

**Figure 3:**
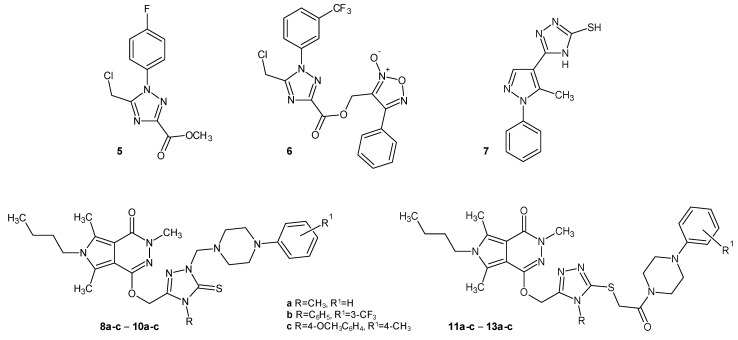
1,2,4-triazole derivatives as COX inhibitors (part 2).

In 2020, Li et al. designed a series of 1,2,4-triazole derivatives with a halogen substituent, and they were examined in vitro for their activity as COX-1/COX-2 inhibitors. Compound **5** (Figure 3) displayed the best COX-2 activity (COX-1 IC_50_ = 19.3 µM and COX-2 IC_50_ = 0.018 µM) and the best selectivity, being three-fold better than celecoxib (COX-1 IC_50_ = 14.8 µM and COX-2 IC_50_ = 0.05 µM). Additionally, in vivo, compound **5** showed greater anti-inflammatory effects and gastric protection at a dose of 5 mg/kg than indomethacin did at a dose of 10 mg/kg. In 2023, the same team headed by Li began testing the potentially most active 1,2,4-triazole derivatives in hybridization with furoxan, and their results showed that these compounds also had anti-inflammatory properties. Compound **6** (Figure 3) showed the best COX-2 inhibitory activity (COX-1 IC_50_ = 9.51 µ and COX-2 IC_50_ = 0.045 µM), which was higher than celecoxib (COX-1 IC_50_ = 8.97 µM and COX-2 IC_50_ = 0.063 µM). In addition, compound **6** was examined in vivo for its pro-inflammatory cytokines production and ulcerogenicity, and it displayed a better inhibition of cytokines and gastric safety compared with indomethacin at the same concentration [18,19].

1,2,4-triazole-pyrazole hybrids acting as COX-1/COX-2 inhibitors were the subject of research by Abdellatif et al. (2021). Their compound **7** (5-(5-methyl -1-phenyl-1*H*-pyrazol-4-yl)-4*H*-1,2,4-triazol-3-thiol) (Figure 3) displayed the highest selectivity index (COX-1/COX-2 ratio) (COX-1 IC_50_ = 593.5 µM and COX-2 IC_50_ = 21.53 µM), and it was four times more effective than celecoxib (COX-1 IC_50_ = 21.53 µM and COX-2 IC_50_ = 3.33 µM). In the in vivo model (carrageenan-induced rat paw edema test), derivative 7 also demonstrated the greatest activity. Additionally, it exhibited cardioprotective effects and was less toxic to gastric cells [20].

In 2021, Szczukowski et al. synthesized new 1,2,4-triazole derivatives of pyrrolo [3,4-*d*]pyridazinone, which were tested for their anti-inflammatory effects. The in vitro results displayed that molecules 8b, 9a, 11a, and 11b (Figure 3) (COX-1 IC_50_ = 70.96–95.75 µM and COX-2 IC_50_ = 47.83–49.79 µM), compared to meloxicam (COX-1 IC_50_ = 83.7 µM and COX-2 IC_50_ = 59.2 µM), showed good inhibitory activity against the COX-2 isoenzyme and were characterized by encouraging the COX-2/COX-1 selectivity ratio. Furthermore, derivatives 8a, 8c, 10a, and 13a demonstrated activity selectively on the COX-2 enzyme (COX-2 IC_50_ = 42.64–48.50 µM), which was supported by molecular docking studies. These compounds also did not show cytotoxicity, and the in vitro tests demonstrated their antioxidant effect on cells [21].

**Figure 4:**
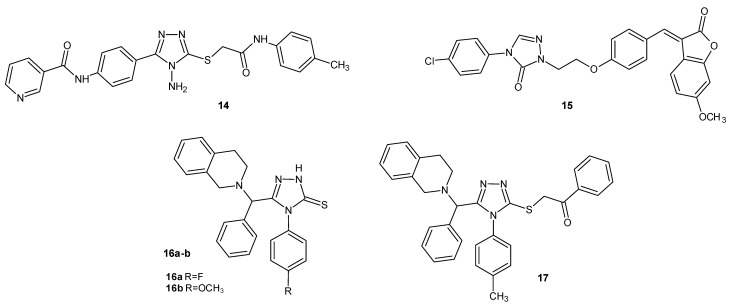
1,2,4-triazole derivatives as COX inhibitors (part 3).

A novel series of 1,2,4-triazole derivatives were designed, synthesized, and their anti-inflammatory effects were established by Hamoud et al. in 2022. The most potent derivative, 14 (Figure 4) (COX-1 IC_50_ = 13.5 µM and COX-2 IC_50_ = 0.04 µM), performed similar to celecoxib (COX-1 IC_50_ = 14.7 µM and COX-2 IC_50_ = 0.045 µM) in its selectivity for COX-2, and it was definitely stronger than diclofenac sodium (COX-1 IC_50_ = 3.8 µM and COX-2 IC_50_ = 0.84 µM). Through the potential to inhibit the production of pro-inflammatory markers (TNF-α and IL-6) and oxidative stress mediators (nitric oxide (NO) and reactive oxygen species (ROS)) in macrophages activated with lipopolysaccharide (LPS), the anti-inflammatory and antioxidant effects of the synthesized compounds were confirmed [22].

The anti-inflammatory effects of 1,2,4-triazole-isoaurone hybrids were the subject of research by Bai’s team (2024). Compound **15** ((*E*)-4-(4-chlorophenyl)-2- (3-(4-((6-methoxy-2-oxobenzofuran-3(2*H*)-ylidene)methyl)phenoxy)propyl)-2,4-dihydro-3*H*-1,2,4-triazol-3-one), shown in Figure 4, exhibited high COX-2 inhibitory activity, with a good selectivity ratio (COX-1 IC_50_ = 18.59 µM and COX-2 I_C50_ = 2.6 µM), and it also inhibited the release of pro-inflammatory factors, including NO and the prostaglandin PGE_2_. Moreover, it was characterized by its low toxicity, and in vivo, in a xylene-induced ear model, it turned out to be the most potent. The anti-inflammatory activity of derivative 15 (41.82%) depended on the dose and was greater than that of celecoxib (31.82%) [23].

Abo-Elmagd et al. (2024) designed a series of 1,2,4-triazole-tetrahydroisoquinoline hybrids as COX-1/COX-2 inhibitors. Compounds **16a**, **16b**, and **17** (Figure 4) were the most impressive derivatives (COX-1 IC_50_ = 2.14–11.95 µM and COX-2 IC_50_ = 0.58–1.27 µM), and they performed better or comparably to the standard drug celecoxib (COX-1 IC_50_ = 15.18 µM, COX-2 IC_50_ = 0.82 µM), but with a decreased selectivity index. During the in vivo carrageenan-induced rat paw edema test, these three compounds consistently showed higher anti-inflammatory activity than celecoxib. Moreover, they were extremely effective in reducing the expression of pro-inflammatory mediators (PGE_2_, TNF-α, and IL-6) and were much safer for stomach cells compared to indomethacin [24].

The next compounds we discuss contain a combination of various pharmacophores that are important for anti-inflammatory activity, as follows: a 1,2,4-triazole ring and sulfamoylphenyl and methylsulfonylphenyl substituents, which occur in selective COX-2 inhibitors (celecoxib and rofecoxib) and are responsible for selectivity towards cyclooxygenase-2.

**Figure 5:**
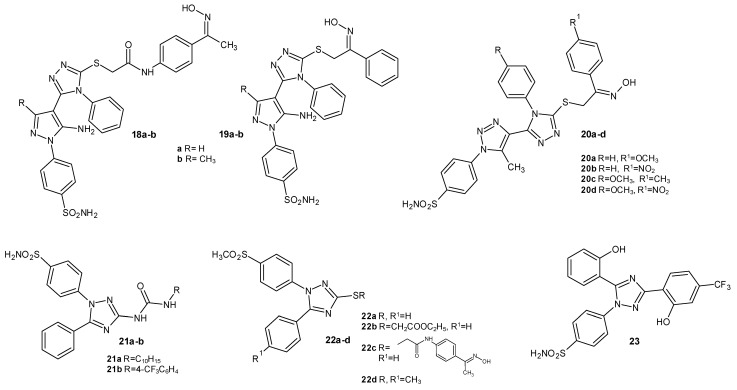
1,2,4-triazole derivatives as COX inhibitors with sulfamoylphenyl or methylsulfonylphenyl substituents.

A new series of 1,2,4-triazole-pyrazole hybrids synthesized by Fadaly et al. in 2020 were tested for their cyclooxygenase inhibitory activity. All tested derivatives were more selective for the COX-2 isoenzyme, especially the sulfamoyl derivatives, such as 18a, 18b, 19a, and 19b (Figure 5) (COX-1 IC_50_ = 5.23–9.81 µM and COX-2 IC_50_ = 0.55–0.91 µM), in comparison to celecoxib (COX-1 IC_50_ = 7.21 µM and COX-2 IC_50_ = 0.83 µM). Additionally, they had better anti-inflammatory activity than celecoxib in vivo and were six times less ulcerogenic than ibuprofen. In 2023, the Fadaly group tested 1,2,4-triazole/1,2,3-triazole hybrids with the sulfamoyl group, and compounds **20a-d** (Figure 5) showed significant selectivity towards COX-2 (COX-1 IC_50_ = 6.08–12.0 µM and COX-2 IC_50_ = 0.24–0.33 µM), performing better than celecoxib (COX-1 IC_50_ = 8.78 µM and COX-2 IC_50_ = 0.42 µM). They also had anticancer activity. All Fadaly’s derivatives were linked to the oxime moiety as nitric oxide donors [25,26].

In 2021, Abdelazeem et al. obtained a series of diaryl-1,2,4-triazole derivatives and determined their inhibition of COX enzymes in vitro. Compounds **21a** and **21b** (Figure 5), with a urea linker and sulfamoylphenyl moiety, were the most potent against COX-2 (COX-1 IC_50_ = 8.85–9.15 µM and COX-2 IC_50_ = 1.98–2.13 µM) in comparison to celecoxib (COX-1 IC_50_ = 6.12 µM and COX-2 IC_50_ = 0.95 µM). In the in vivo assays, they also exhibited greater analgesic and anti-inflammatory effects than celecoxib. Derivatives 21a and 21b also exhibited activity against sEH (soluble epoxide hydrolase). This enzyme inactivates epoxyeicosatrienoic acid (EET), which has anti-inflammatory, anti-aggregation, and vasodilatation effects. The inhibition of sEH allows for a strengthened cardioprotective effect. Compounds with dual COX-2/sEH inhibitory activity are expected to have fewer adverse effects on the cardiovascular system [27].

In 2022, Abdellatif’s team synthesized 1,2,4-triazole derivatives containing a methylsulfonylphenyl group interacting with the COX enzyme. Compounds **22a-d**, presented in Figure 5, showed the highest selectivity towards COX-2 in the in vitro assay (COX-1 IC_50_ = 76.62–140.9 µM and COX-2 IC_50_ = 5.25–10.17 µM) in comparison to celecoxib (COX-1 IC_50_ = 21.48 µM and COX-2 IC_50_ = 3.33 µM). They also displayed significant activities in vivo and a lower ulcerogenic potential than celecoxib and indomethacin [28].

In 2023, Wang et al. conducted research aimed at examining the neuroprotection of synthesized compounds through their anti-inflammatory activity. 1,3,5-triphenyl-1,2,4-triazole compounds were synthesized, and their selectivity towards COX-2 was verified. The most potent compound turned out to be derivative **23** (Figure 5), with a benzenesulfonamide moiety, as it had the best selectivity index (COX-1 IC_50_ = 86.52 µM and COX-2 IC_50_ = 0.54 µM) among all the synthesized compounds in comparative test with celecoxib (COX-1 IC_50_ = 26.46 µM and COX-2 IC_50_ = 0.092 µM). Also, it had a neuroprotective effect in inhibiting the pathways of oxidative stress, inhibiting COX-2 and inducible nitric oxide synthase (iNOS) expression, and reducing levels of NO and PGE_2_. Additionally, this compound had less cardiotoxicity than celecoxib [29].

### 2.2. 1,2,4-Triazole Derivatives as Lipoxygenase Inhibitors

In addition to structures that inhibit cyclooxygenases, 1,2,4-triazole derivatives with lipoxygenase inhibitory activity have been reported as promising anti-inflammatory compounds, and they are further examined in this section. First, 15-LOX inhibitors are discussed, followed by 5-LOX inhibitors and bifunctional compounds that inhibit COX-2 and 5-LOX.

Lipoxygenases (LOXs) are responsible for many functions such as cell differentiation, formation of the skin barrier, and immunity. LOXs play a crucial role in the biosynthesis of active pro-inflammatory mediators like leukotrienes from arachidonic acid, which further demonstrate potent effects on the aggregation of leukocytes, the contraction of smooth muscles, and vascular permeability that, as a consequence, contributes to inflammatory diseases like rheumatoid arthritis, asthma, psoriasis, and inflammatory bowel disorders. The various types of mammalian LOX enzymes display diverse biological functions. Pro-inflammatory leukotrienes (LTs) are synthesized through 5-LOX activity. On the other hand, anti-inflammatory lipoxins can be synthesized by the connected activities of 15-LOX and 5-LOX. By blocking those enzymes, it is possible to reduce inflammation and limit its negative effects on the body [30,31].

#### 2.2.1. 1,2,4-Triazole Derivatives as 15-LOX Inhibitors

**Figure 6:**
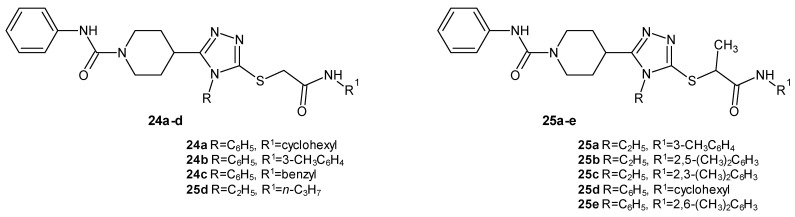
1,2,4-triazole derivatives as 15-LOX inhibitors (part 1).

A team of researchers led by Muzaffar (2020–2021) synthesized two series of hybrids of phenylcarbamoylpiperidine and 1,2,4-triazole acetamide derivatives with phenyl or ethyl substituents in position four of the 1,2,4-triazole ring, and they were investigated for their inhibitory potential of the 15-lipooxygenase enzyme in comparison to the reference drug quercetin (IC_50_ = 4.86 µM). The series with a phenyl substituent was more active, and the IC_50_ values for the strongest derivatives (**24a**, **24b**, and **24c** (Figure 6)) ranged from 9.25 to 13.64 µM, while for the series with an ethyl substituent, the most active derivative, **24d** (Figure 6), showed an IC_50_ of 17.52 µM while maintaining low toxicity for all potent compounds [31,32].

The studies by Nawaz et al. (2024) were a continuation of the above-mentioned research by Muzaffar. The team synthesized hybrids of substituted phenylcarbamoylpiperidine and 1,2,4-triazole methylacetamide derivatives with phenyl or ethyl substituents in position four of the 1,2,4-triazole ring in search of potent 15-LOX inhibitors. The inhibitory potential of the 15-lipooxygenase enzyme was investigated in comparison to the reference drugs quercetin (IC_50_ = 4.86 µM) and baicalein (IC_50_ = 2.24 µM). The derivatives with an ethyl substituent were more active, and the strongest compounds (**25a**, **25b**, and **25c** (Figure 6)) displayed extraordinary potency, with IC_50_ values of below 1 µM (0.36–0.84 µM), while the most active derivatives with a phenyl substituent (25d and 25e (Figure 6)) showed activity at the IC_50_ level of 1.02–3.39 µM. The most outstanding derivatives were characterized by low toxicity [33,34].

Summarizing the work of Muzzafar and Nawaz, it is possible to conclude that the amide bond is essential for LOX inhibition. Also, each part of the compound (the phenylcarbamoylpiperidine moiety and 1,2,4-triazole scaffold) is responsible for the appropriate bonds with the enzyme, and the hydrophobic terminals enhance lipophilicity.

**Figure 7:**
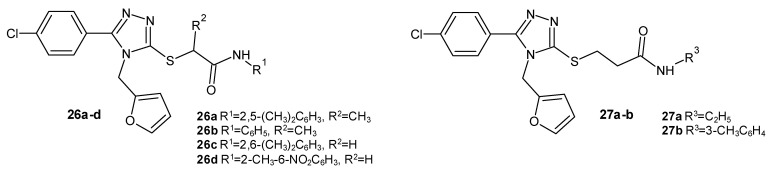
1,2,4-triazole derivatives as 15-LOX inhibitors (part 2).

In 2022–2023 Yasin and Riaz et al. synthesized new *N*-furfurylated 4-chlorophenyl-1,2,4-triazole acetamide, methylacetamide, and propionamide derivatives to find significant 15-lipoxygenase inhibitors. The inhibitory potential of the 15-LOX enzyme was investigated in comparison to the reference drugs quercetin (IC_50_ = 4.86 µM) and baicalein (IC_50_ = 2.24 µM). The most active and the least toxic, although much weaker than the standard drugs, were the methylacetamide derivatives 26a and 26b (IC_50_ = 17.43–19.35 µM), as presented in Figure 7. The most active acetamide derivatives, 26c and 26d (Figure 7), had IC_50_ values of between 19 and 28 µM. The weakest but also the most toxic were the propionamide derivatives 27a and 27b (IC_50_ = 21.83–25.72 µM) (Figure 7) [35,36,37].

#### 2.2.2. 1,2,4-Triazole Derivatives as FLAP Inhibitors

**Figure 8:**
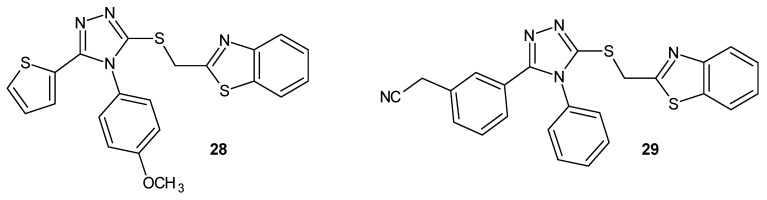
1,2,4-triazole derivatives as FLAP inhibitors.

Olgac et al. (2023) examined substituted 1,2,4-triazoles as innovative and selective inhibitors of 5-lipoxygenase-activating protein (FLAP). The inspiration for the synthesis of the derivatives was compound **28** (Figure 8) selected in a multistep virtual screening protocol undertaken 3 years earlier, which managed to pick up several of the potentially most active FLAP inhibitors among millions of compounds. Cellular leukotriene biosynthesis is regulated by FLAP, which controls the transmission of arachidonic acid (AA) to 5-lipoxygenase for effective metabolism. The most potent compound, **29** (IC_50_ = 1.15 µM) (Figure 8), exhibited twice the activity of 28 (IC_50_ = 2.18 µM). Applying human macrophages and neutrophils, the researchers proved that derivative **29** displayed suppression of the formation of 5-lipooxygenase products (5-hydroxyeicosatetraenoic acid (5-HETE), leukotriene LTB_4_, and its all-trans isomers) as a result of the antagonism of FLAP, without the participation of other enzymes such as COX, 12/15-LOX, and cytochrome P450 [38,39].

#### 2.2.3. 1,2,4-Triazole Derivatives as COX-2/5-LOX Inhibitors

**Figure 9:**
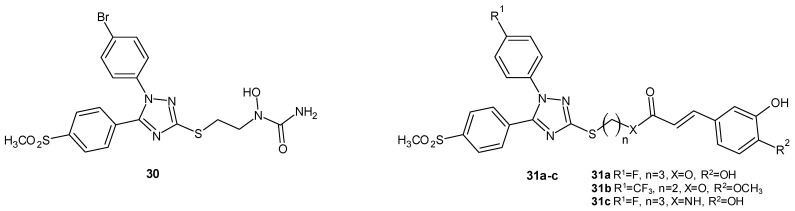
1,2,4-triazole derivatives as COX-2/5-LOX inhibitors (part 1).

Jiang et al. (2014) searched for anti-inflammatory molecules by obtaining a series of diaryl-1,2,4-triazoles containing an *N*-hydroxyurea moiety, and these served double inhibitors of cyclooxygenase-2 and 5-lipoxygenase. The reference drugs were celecoxib (COX-2 IC_50_ = 0.14 µM) and zileuton (5-LOX IC_50_ = 0.82 µM). The strongest compound, **30** (1-(2-(1-(4-bromophenyl)-5-(4-(methylsulfonyl)phenyl)-1*H*-1,2,4-triazol-3-ylthio)ethyl)-1- hydroxyurea), as presented in Figure 9, showed COX-2 and 5-LOX inhibitory activities, very similar to the reference drugs (COX-2 IC_50_ = 0.15 µM and 5-LOX IC_50_ = 0.85 µM). Derivative 30 exhibited an in vivo effectiveness comparable to celecoxib in the xylene-induced ear edema assay, and it also demonstrated analgesic activity [40].

Cai’s team (2016) obtained novel hybrids of diaryl-1,2,4-triazoles and caffeic acid (CA) as dual COX-2/5-LOX inhibitors. The derivatives were examined in vitro in comparison to the reference drugs celecoxib (COX-2 IC_50_ = 0.14 µM) and zileuton (5-LOX IC_50_ = 0.80 µM). The activities of the compounds **31a** and **31b** (Figure 9) were comparable with the standard drugs (COX-2 IC_50_ = 0.18–0.21 µM and 5-LOX IC_50_ = 0.81 µM), but the most potent among all the derivatives was 31c (COX-2 IC_50_ = 0.12 µM and 5-LOX IC_50_ = 0.71 µM), which was even more active than celecoxib and zileuton. In addition, compounds **31a** and **31b** showcased anti-cancer activity. By comparing the structure–activity relationships of these hybrid derivatives, it could be concluded that the electron-withdrawing substituents (Br, F, and CF_3_) enhanced the activity of the new compounds, and a methylsulfonyl substituent was required [41].

**Figure 10:**
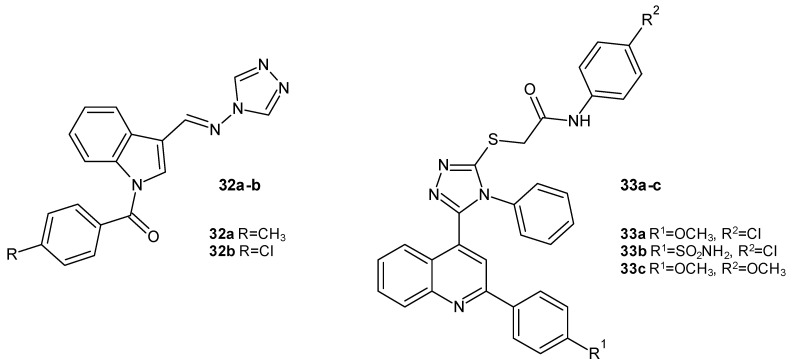
1,2,4-triazole derivatives as COX-2/5-LOX inhibitors (part 2).

Lamie et al. (2016) developed a novel series of *N*-substituted indole Schiff bases as double inhibitors of cyclooxygenase-2 and 5-lipoxygenase. Compounds **32a** and **32b** (Figure 10), bearing 1,2,4-triazole rings, exhibited remarkably great COX-2 inhibitory activity (IC_50_ = 0.98–1.23 µM) in comparison to the standard drug celecoxib (IC_50_ = 1.54 µM), and they displayed improved COX-2 selectivity indexes. These compounds also inhibited 5-LOX (IC_50_ = 6.54–8.11 µM) but were weaker than the reference drug quercetin (5-LOX IC_50_ = 5.96 µM) [42].

In 2021, Mohassab et al. determined the anti-inflammatory effects of a new series of quinolone-1,2,4-triazole hybrids as dual COX-2/5-LOX inhibitors. Compounds **33a**, **33b**, and **33c** (Figure 10) exhibited the greatest potencies and selectivities in inhibiting COX-2 (COX-2 IC_50_ = 7.25–8.48 nM) in comparison to celecoxib (COX-2 IC_50_ = 42.60 nM). Compound **33a** was also the most potent 5-LOX inhibitor (5-LOX IC_50_ = 5.43 µM), corresponding to the value of the standard drug quercetin (5-LOX IC_50_ = 5.96 µM), while the other two derivatives were three times weaker. The anti-inflammatory activity of **33a-c** was confirmed in vivo by applying the carrageenan-induced paw edema test. These compounds were also definitively safer for stomach cells than indomethacin. Additionally, compounds **33a-c** significantly reduced the expression of pro-inflammatory cytokines (PGE_2_, TNF-α, and IL-6) [43].

### 2.3. 1,2,4-Triazole Derivatives with Confirmed Anti-Inflammatory Activity In Vivo

In addition to articles describing compounds tested in vitro for enzyme activity (COX/LOX inhibition), there is also a large group of derivatives containing the 1,2,4-triazole ring which were tested only in in vivo models, and they have shown anti-inflammatory effects and are discussed in this section.

**Figure 11:**
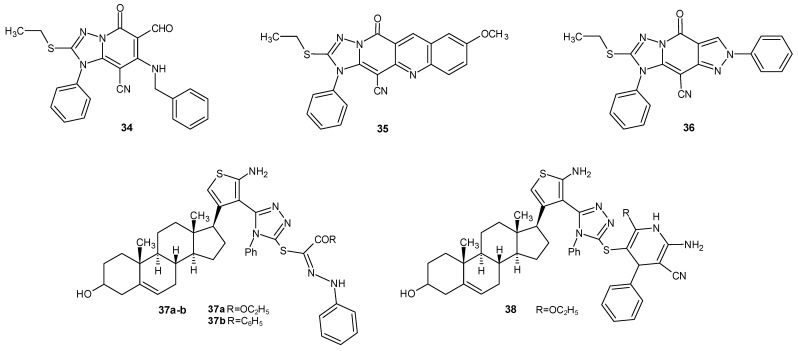
1,2,4-triazole derivatives with confirmed anti-inflammatory activity in vivo (part 1).

A team of researchers under the direction of Mekheimer (2015) synthesized a series of 1,2,4-triazolo [1,5-*a*]pyridines and their fused ring systems. In the in vivo studies, based on the inflammation induced by carrageenan and dextran, compounds **34**, **35**, and **36** (Figure 11) exhibited significant anti-inflammatory properties comparable to the standard drug indomethacin. Compounds **34** and **35** also demonstrated antioxidant properties, with high radical scavenging activity [44].

The anti-inflammatory activity of newly synthesized pregnenolone derivatives with thiophene and 1,2,4-triazole ring substituents was investigated by a team led by El-Sayed in 2016. In the in vivo studies on carrageenan-induced rat hind paw edema, compounds **37a**, **37b**, and **38**, presented in Figure 11, showed significant anti-inflammatory properties (derivative **38** displayed the same activity as the reference drug indomethacin). It is noteworthy that they were non-toxic, and compound **38** demonstrated the weakest antiulcer activity [45].

**Figure 12:**
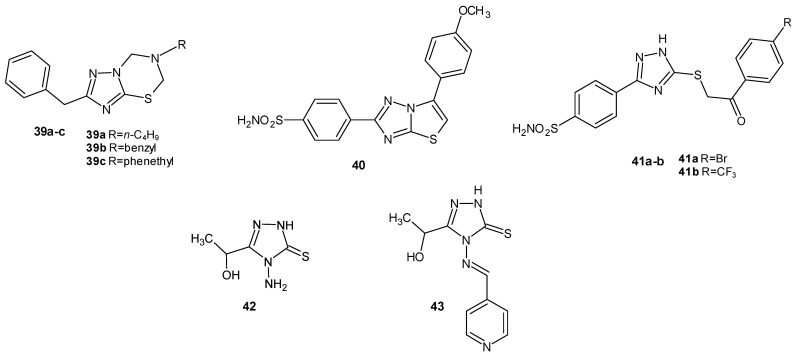
1,2,4-triazole derivatives with confirmed anti-inflammatory activity in vivo (part 2).

A team of researchers from Turkey (Sert-Ozgur et al., 2017) synthesized condensed 1,2,4-triazolo [3,2-*b*]-1,3,5-thiadiazine derivatives to obtain compounds with anti-inflammatory and analgesic properties. In vivo carrageenan-induced rat hind paw edema studies were conducted to assess the anti-inflammatory properties. Based on the research findings, it was concluded that the compounds carrying the benzyl group at the second position were the most promising compounds, such as **39a-c** (Figure 12). Compared to the reference drug naproxen, they achieved comparable anti-inflammatory values while maintaining reduced antiulcer activity [46].

Cristina et al. (2018) synthesized a series of thiazolo [3,2-*b*]-1,2,4-triazoles and their corresponding acyclic intermediates bearing a benzenesulfonamide moiety. In the in vivo study on the anti-inflammatory activity of the synthesized compounds on carrageenan-induced paw edema in rats, compounds **40**, **41a**, and **41b**, as presented in Figure 12, exhibited the most significant anti-inflammatory activity in comparison to diclofenac, which was the reference drug. In the study, all compounds demonstrated significantly lower ulcerogenic properties compared to diclofenac [47].

A team led by Azim (2021) conducted an in vivo evaluation of the anti-inflammatory, analgesic, and antipyretic activities of newly synthesized 1,2,4-triazole derivatives. In a comparative in vivo study (carrageenan-induced paw edema in mice) with the standard drug ibuprofen, compound **42** (Figure 12) demonstrated a 91% level of activity compared to 82% for ibuprofen, while compound **43** (Figure 12) exhibited properties similar to the reference drug (81%). The derivatives were also assessed for their anti-nociceptive activity using the acetic acid writhing and tail immersion tests. In this study, compound **42** showed a reduction in writhing at a level of 81%, while compound **43** showed a level of 70%, which was comparable to the values achieved by ibuprofen (71.5%). No toxicity was observed for the synthesized derivatives in the in vivo study [48].

**Figure 13:**
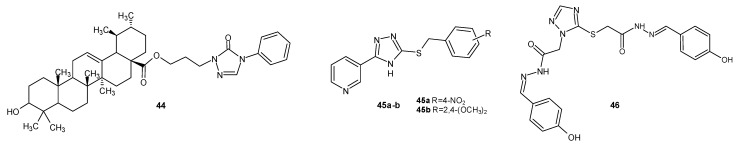
1,2,4-triazole derivatives with confirmed anti-inflammatory activity in vivo (part 3).

In 2018, researchers from China under the direction of Wei et al. synthesized derivatives of ursolic acid containing different heterocyclic rings. In vivo studies on a para-xylene-induced mice ear-swelling model revealed that compound **44**, with a 1,2,4-triazol-5(4*H*)-one moiety, as presented in Figure 13, exhibited the greatest anti-inflammatory properties. Comparative tests with ibuprofen and indomethacin showed that 44 was stronger than the reference drugs and did not exhibit toxicity. Docking studies also were performed, and compound **44** demonstrated a high affinity for the active center of COX-2 [49].

Ahirwar et al. (2018) developed compounds containing a 1,2,4-triazole ring and substituted benzyl groups via thio linkage, and they investigated their anti-inflammatory activity. The in vivo analgesic (acetic acid-induced writhing reflex) and anti-inflammatory (carrageenan-induced paw edema) studies showed the significant activities of **45a** and **45b** (Figure 13). Docking studies against COX-2, which were additionally performed, revealed that compound **45a** exhibited a binding model with the COX-2 chain A that was most similar to acetylsalicylic acid (ASA). In contrast, compound **45b** resembled the binding model of indomethacin, with the COX-2 chain B [50].

Khan’s team (2021) developed a series of 1,2,4-triazole derivatives and investigated their anti-inflammatory activity. In vivo, carrageenan-induced rat paw edema studies showed that the most active derivative, 46 (Figure 13), exhibited activity similar to the standard drug indomethacin. Compound **46** also showed antioxidative free radical scavenging activity. Moreover, molecular docking studies showed that 46 occupied the celecoxib binding site in cyclooxygenases, with high affinity [51].

### 2.4. 1,2,4-Triazole Derivatives with Miscellaneous Mechanisms for Anti-Inflammatory Activity

The last section discussed new compounds containing a 1,2,4-triazole scaffold with various mechanisms of anti-inflammatory action, and it did not include the previously mentioned molecules. Some derivatives influence the level of pro-inflammatory cytokines, while others act by changing the activities of enzymes different than COX/LOX.

**Figure 14:**
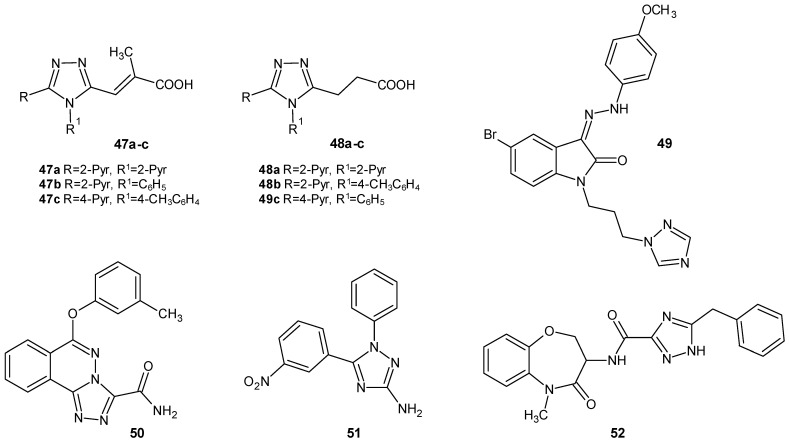
1,2,4-triazole derivatives with miscellaneous mechanisms for anti-inflammatory activity (part 1).

A team of researchers led by Paprocka over a period of several years (2015–2023) synthesized a series of 1,2,4-triazole derivatives substituted with methacrylic acid, 2-methyl acrylic acid, and propionic acid. The influence of the newly obtained compounds on inflammation and on the level of cytokine release (TNF-α, IL-6, IL-10, and interferon-γ (IFN-γ)) in lipopolysaccharide (LPS) -stimulated peripheral blood mononuclear cells (PBMC) was experimentally evaluated. Compounds **47a-c** (Figure 14) from the series of derivatives of 2-methyl acrylic acid, as well as derivatives **48a-c** (Figure 14) from the series of propionic acid derivatives, were considered to be the most active. They inhibited the production of cytokines to the greatest extent. All of them significantly reduced the level of TNF-α and decreased the release of IFN-γ, and they were additionally characterized by their low toxicity [52,53,54].

In 2016, Sharma et al. published the results of their research on 1,2,4-triazole-indolin-2-one hybrids with anti-inflammatory effects. The obtained compounds were tested for their inhibitory activity on the TNF-α-induced expression of intercellular adhesion molecule-1 (ICAM-1) on the surface of human umbilical vein endothelial cells (HUVEC). Increased levels of adhesion molecules on endothelial cells alter the adhesive character of the vascular system, and this leads to the extensive infiltration of leukocytes, thereby causing inflammation. The strongest derivative, 49 (*Z*-1-[2-(1*H*-1,2,4-triazol-1-yl)propyl]-3-[2-(4-methoxyphenyl)hydrazono]indolin-2-one), as presented in Figure 14, inhibited ICAM-1 expression by 89%, with an IC_50_ value of 20 mM at a maximum tolerated dose of 200 mM in HUVECs [55].

In 2016, Liu et al. designed and synthesized 6-phenoxy-1,2,4-triazolo [3,4-*a*]phthalazine-3-carboxamide derivatives and studied their anti-inflammatory activity. Compound **50** (Figure 14) exhibited significant activity as an inhibitor of the activation of nuclear factor kappa B (NF-κB) transcription factors, comparable to the reference drug dihydrotanshinone. These factors are integral to the immune response and are thus capable of indirectly regulating the inflammatory process in the body. In an in vivo anti-inflammatory test, 50 exhibited excellent activity, with an efficacy equal to indomethacin. Additionally, compound **50** displayed the lowest toxicity among the tested compounds [56].

Grewal et al. (2017) obtained a series of 1,2,4-triazole derivatives and examined their ability to inhibit the activity of phosphodiesterase 4 (PDE4). PDE4 belongs to the group of phosphodiesterase enzymes, which catalyzes the hydrolysis of cyclic adenosine monophosphate to adenosine monophosphate in immunomodulatory and pro-inflammatory cells, which leads to expanded inflammatory processes. The novel compounds were tested in silico through docking studies to determine their PDE4 protein-binding site interactions. In vivo, the anti-inflammatory effects of the most active derivatives were confirmed using a carrageenan-induced paw edema assay. Compound **51** ((5-(3-nitrophenyl)-1-phenyl-1*H*-1,2,4-triazole-3-amine) (Figure 14) demonstrated the most notable activity compared to ibuprofen [57].

Harris et al. (2017) researched receptor-interacting protein kinase 1 (RIPK1) inhibitors. RIPK1 is responsible for inflammation and necroptosis, and thus, it may influence miscellaneous pathologies, including immune-mediated inflammatory diseases. Tumor necrosis factor signaling through the RIPK1 pathway regulates colitis, and therefore, the inhibition of RIPK1 may be a potential beneficial goal for ulcerative colitis (UC) treatment. The most active compound, **52** (Figure 14), called GSK2982772, was a first-in-class oral and selective RIPK1 inhibitor that would bind to the allosteric pocket of the RIPK1 kinase domain to inhibit RIPK1-mediated cytokine production and cell death. Multiple clinical trials are currently underway using GSK2982772 in patients with psoriasis, rheumatoid arthritis, and UC [58,59,60].

**Figure 15:**
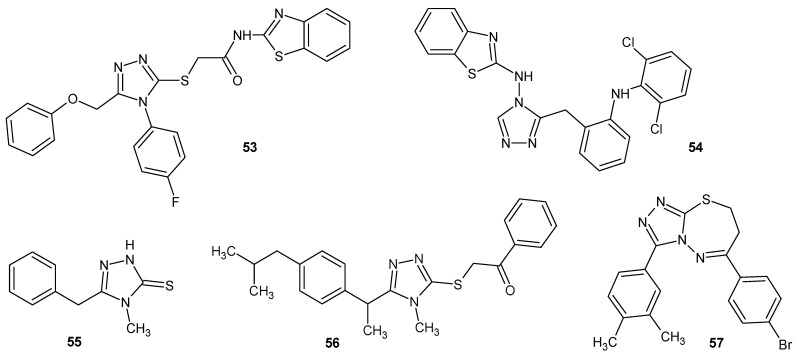
1,2,4-triazole derivatives with miscellaneous mechanisms for anti-inflammatory activity (part 2).

Tariq et al. (2018) obtained two series of 1,2,4-triazole derivatives containing benzothiazole rings. The compounds were found to have anti-inflammatory effects in the in vitro and the in vivo tests. The most active compounds, **53** and **54**, as presented in Figure 15, were stronger than the reference drug diclofenac sodium, and they were also characterized by their lower ulcerogenic potential and protective effect on lipid peroxidation. Additionally, in searching for a probable mechanism of action, a p38α mitogen-activated protein (MAP) kinase assay was performed, which revealed the high inhibitory potential of the kinases of both compounds, and molecular docking studies confirmed their high affinity for MAPK. The p38α MAP kinase (MAPK) significantly influences the inflammatory processes. The α-isoform exhibits a crucial role in the biosynthesis of proinflammatory cytokines such as tumor necrosis factor (TNF-α) and interleukin (IL-1β). The inhibition of MAPK enhances anti-inflammatory effects and, in some reports, also suppresses COX-2 expression [61,62].

Researchers from Turkey (Bülbül et al., 2023) developed a series of 1,2,4-triazoles derived from ibuprofen. Their impact on the activity of microsomal prostaglandin E_2_ synthase-1 (mPGES-1) was examined because of the collaboration of COX-2 and mPGES-1 enzymes in the production of PGE_2_. According to the results, compounds **55** and **56** (Figure 15), characterized by their low cytotoxicity, demonstrated good anti-inflammatory activity against the mPGES-1 enzyme, which was confirmed by molecular docking studies. Additionally, their COX inhibitory activity was verified. Both compounds inhibited COX-2 more strongly than they inhibited COX-1. In addition, their anticancer effects and ability to inhibit angiogenesis were tested [63].

Researchers under the direction of Erdogan in 2024 synthesized a series of 1,2,4-triazolo[3,4-*b*][1,3,4]thiadiazine derivatives and tested them for inhibition abilities of releasing pro-inflammatory factors (NO, PGE_2_, and IL-6). All of the compounds were observed to reduce LPS-induced nitric oxide production in a concentration-dependent manner. The most active with the lowest dose (1 μM) was compound **57** (6-(3,4-dimethylphenyl)-3-(4-bromophenyl)-7*H*-1,2,4-triazolo [3,4-*b*][1,3,4]thiadiazine) (Figure 15), which exhibited a 62% inhibition of NO production, while indomethacin showed a 52% inhibition at 100 μM. Also, the derivatives were evaluated for their in vitro effects on PGE_2_, IL-6, and iNOS levels in murine macrophage cells stimulated with LPS. Derivative 57 at the lowest dose (1 μM) displayed a comparable activity to indomethacin at a dose of 100 μM [64].

## 3. Summary

The conducted review of 1,2,4-triazole derivatives demonstrates their anti-inflammatory activity. These compounds differ in their mechanisms of action, which could lead to new therapeutic approaches compared to currently available drugs. Some of the presented derivatives in the review showed in vivo activity. Moreover, the 1,2,4-triazole derivatives in the in vitro tests acted as inhibitors of COX-1/COX-2 and LOX, and they also exhibited distinct mechanisms of action such as the inhibition of pro-inflammatory cytokine release or interactions with other enzymes, e.g., phosphodiesterase 4, different protein kinases, or prostaglandin synthase. The most active compounds showed greater efficacy than the reference drugs used in the studies, highlighting their promising potential as new pharmacological solutions. Further in vivo studies, especially for compounds that have only undergone in vitro or molecular docking studies, are necessary to confirm their effectiveness, activity, and safety.

## Data Availability

In this review article, no original data were reported.
